# Disease Classification Based on Eye Movement Features With Decision Tree and Random Forest

**DOI:** 10.3389/fnins.2020.00798

**Published:** 2020-08-06

**Authors:** Yuxing Mao, Yinghong He, Lumei Liu, Xueshuo Chen

**Affiliations:** State Key Laboratory of Power Transmission Equipment & System Security and New Technology, Chongqing University, Chongqing, China

**Keywords:** eye movement, disease discrimination, decision tree, random forest, long short-term memory

## Abstract

Medical research shows that eye movement disorders are related to many kinds of neurological diseases. Eye movement characteristics can be used as biomarkers of Parkinson’s disease, Alzheimer’s disease (AD), schizophrenia, and other diseases. However, due to the unknown medical mechanism of some diseases, it is difficult to establish an intuitive correspondence between eye movement characteristics and diseases. In this paper, we propose a disease classification method based on decision tree and random forest (RF). First, a variety of experimental schemes are designed to obtain eye movement images, and information such as pupil position and area is extracted as original features. Second, with the original features as training samples, the long short-term memory (LSTM) network is used to build classifiers, and the classification results of the samples are regarded as the evolutionary features. After that, multiple decision trees are built according to the C4.5 rules based on the evolutionary features. Finally, a RF is constructed with these decision trees, and the results of disease classification are determined by voting. Experiments show that the RF method has good robustness and its classification accuracy is significantly better than the performance of previous classifiers. This study shows that the application of advanced artificial intelligence (AI) technology in the pathological analysis of eye movement has obvious advantages and good prospects.

## Introduction

Eye movement information can be used as an important indicator of a variety of medical diseases and has been a focus of extensive research. Summarizing recent academic literature, the correlations between eye movement and congenital nystagmus, autism, depression, schizophrenia, Parkinson’s syndrome, vertigo, and epilepsy have been verified and studied.

Yang et al. (2015) developed a digital eye tracker to determine the efficacy of congenital idiopathic nystagmus surgery. Giordano et al. (2017) proposed a computer system for ophthalmologists and neuropsychiatrists to customize experiments according to different scenarios and employed it in investigation experiments for patients with glycogen storage disease, idiopathic congenital nystagmus, and neurodevelopmental diseases to assess their eye movement performances.

Murias et al. (2018) carried out an eye gaze tracking experiment in which children with autism spectrum disorder (ASD), aged 24–72, months were asked to watch a videotape that was designed to attract the attention of children to evaluate their social communication skills. They analyzed the relationship between the children’s eye gaze performance and social communication outcome measures that are typically used in ASD clinical trials, and it was proposed that eye gaze tracking could be a non-invasive, quantitative, and objective biomarker associated with social communication abilities in children with ASD. Vargas-Cuentas et al. (2017) designed an eye tracking algorithm to measure the gaze preferences of children with ASD and of a healthy control group when viewing social scenes and abstract scenes shown simultaneously on the left and the right sides of the screen, respectively, and achieved very high accuracy in the classification of children with ASD and healthy children. Dicriscio and Troiani (2017) implemented a paradigm designed to capture patterns of pupil adaptation in children with ASD during sustained periods in dark and light environments. It was found that pupil dilation features are related to individual differences measured by the Social Responsiveness Scale, a measurement for autism traits.

Typical applications of eye movement information in the diagnosis of depression have been described in previous papers (Li et al., 2016; Shengfu et al., 2017; Xu et al., 2017a; Duque and Vazquez, 2018). Shengfu et al. (2017) had patients with major depressive disorder (MDD) and non-depressed controls complete eye tracking tasks and analyzed the attention preference of the participants according to positive, negative, and neutral expressions. According to the results of their study, eye performance in free observation tasks can also be affected by age. Malsert et al. (2012) carried out an experiment in which patients with MDD performed an antisaccade task, and the results suggested that antisaccade performance is associated with the clinical scale score. Furthermore, because error rates had good performance in predicting patients’ states after depressive disorder therapies, error rates could be a state marker for mood disorders (Malsert et al., 2012).

Research on the diagnosis of schizophrenia has also involved eye movement analysis (Dowiasch et al., 2016; Yu et al., 2016; Morita et al., 2017; Xu et al., 2017b; Silberg et al., 2018). Morita et al. (2017) considered eye movements as a biomarker of schizophrenia. They recruited 85 schizophrenia patients and 252 healthy controls to perform free fixation, stable fixation, and smooth tracking tasks and employed an integrated eye movement score to distinguish patients with schizophrenia from healthy controls. Asgharpour et al. (2015) examined the relationship between symptom severity and visual attention allocation while facing emotion-neutral face pairs between adult patients with schizophrenia and healthy controls and concluded that the facial recognition deficit of schizophrenia is related to decreased attention to face stimuli.

Some scholars have applied eye movement information to Alzheimer’s disease (AD) research (Coubard, 2016; Fernández et al., 2016; Lim et al., 2016). Lim et al. (2016) analyzed cerebrospinal defects through fluid analysis, brain imaging, and postmortem, which are commonly applied methods for detecting AD pathological biomarkers, and presented the evidence of ocular biomarkers in AD to explore potential future research approaches of eye movement analysis for AD diagnosis.

As a type of dyskinesia, Parkinson’s disease is likely to be related to optic nerve dysfunction, which affects the cerebral cortex and the subcortical network due to neurodegeneration, causing abnormal eye movements. Archibald et al. (2013) carried out an experiment in which a series of visuo-cognitive tasks was performed by patients with Parkinson’s disease, with and without cognitive impairment, and the error rates and the visual exploration strategies of the patients were examined. According to their study, the visual performances of patients are impaired significantly regardless of cognitive damage, which indicates that there could be a disease-specific impact on the networks directing visual search or exploration.

Wiener-Vacher et al. (2019) gave various oculomotor tasks before and after orthoptic vergence training to children who had dizziness with vergence disorders and found that their orthoptic and oculomotor parameters improved significantly after the training. Degirmenci et al. (2010) performed tests including tracking, saccade, optokinetic, gaze, positional, and Dix–Hallpike tests on patients with vertigo and/or other problems related with equilibrium caused by relapsing–remitting multiple sclerosis (RRMS) and found electronystagmographical characteristics sensitive to detecting the vestibular system involvement in RRMS patients.

Hunfalvay et al. (2019) carried out research focused on people with different severities of traumatic brain injury (TBI) as well as asymptomatic controls. Eye tracking tests were performed to measure horizontal and vertical saccades, and it was concluded that eye tracking methods could be a reliable way to quantify the severity of TBI. Reddy et al. (2019) measured the parameters of eye movement while reading in subjects with TBI and found the parameters to be affected by TBI no matter the severity of the injury, compared to controls. Vakil et al. (2019) presented TBI patients and healthy controls with photographs of male faces, and the result showed that TBI patients paid less attention to the given target and had less dwell time on them, and their memory for faces was also impaired.

Concussion is a form of mild TBI incurred through direct or transmitted impulses to the head that result in functional brain injury. Video-oculographic (VOG) recordings of eye movements can quantitatively describe ocular motor performance in concussed subjects (Williams et al., 1997; Crevits et al., 2000; Heitger et al., 2002, 2004, 2006, 2009; Kraus et al., 2007; Pearson et al., 2007; Rizzo et al., 2016). Through investigation, VOG was found to be an objective and precise way for acquiring eye movement data for concussion evaluation, although some issues including cost, availability, and explanation still need to be explored (Akhand et al., 2019).

Artificial intelligence (AI) is increasingly delivering breakthroughs in numerous research fields and is urgently demanded in disease diagnosis. Considering that eye movement can be recorded and collected conveniently in the form of video, some machine learning methods are especially suitable for dealing with eye movement videos, including long short-term memory (LSTM) network, decision tree, and random forest (RF).

Kothari et al. (2020) trained and evaluated RF and a recurrent neural network (RNN) model to develop algorithms for the categorization of gaze events (e.g., fixations, pursuits, saccade, and gaze shifts) without fixing the head. Zandi et al. (2019) performed a driving simulation experiment to measure driving behavior for detection of drowsiness. The eye tracking data were input into an RF and a non-linear support vector machine for binary classification of the state of vigilance, and the RF method achieved an accuracy of ∼90%.

According to investigation in published papers, machine learning methods are promising ways of handling eye videos, and many fields have already benefited from them. Up to now, there have been few reports of machine learning being used in eye movement analysis and disease diagnosis. They are usually analyzed in traditional ways, which may limit the mining of eye movement features and neglect some valuable information.

Besides that, to better excavate eye movement features, multiple experimental schemes should be designed and, after collecting eye videos, eye movement information should be extracted and quantified to obtain appropriate medical characteristics.

Artificial intelligence research confirms that deep learning can automatically acquire valuable features and improve the robustness of object classification by learning a large number of samples. Thus, AI techniques can be adopted to build eye movement-based disease classifiers. In this paper, experiments are designed to obtain eye videos, and then multiple original eye movement features are extracted for further analysis. The original eye movement features are used to build the LSTM classifiers, and then the output probabilities of the samples are used as evolutionary features for subsequent decision tree and RF construction. The classification results are eventually obtained with the RF.

## Materials and Methods

### Experimental Scheme Design and Original Eye Movement Features Extraction

#### Experimental Scheme Design

When human eye activity is used in related scientific research, the first step is to record and extract eye movement information. The experimental scheme used in this study was designed to generate natural or synthetic images on the display screen, guide eye gazing or tracking, and record eye images at the same time. Through image analysis, the position, area, shape, and other pupil information were obtained. Medical characteristics can then be obtained by mining the pupil information and can be applied for physiological lesions and psychological state analysis.

**FIGURE 1:**
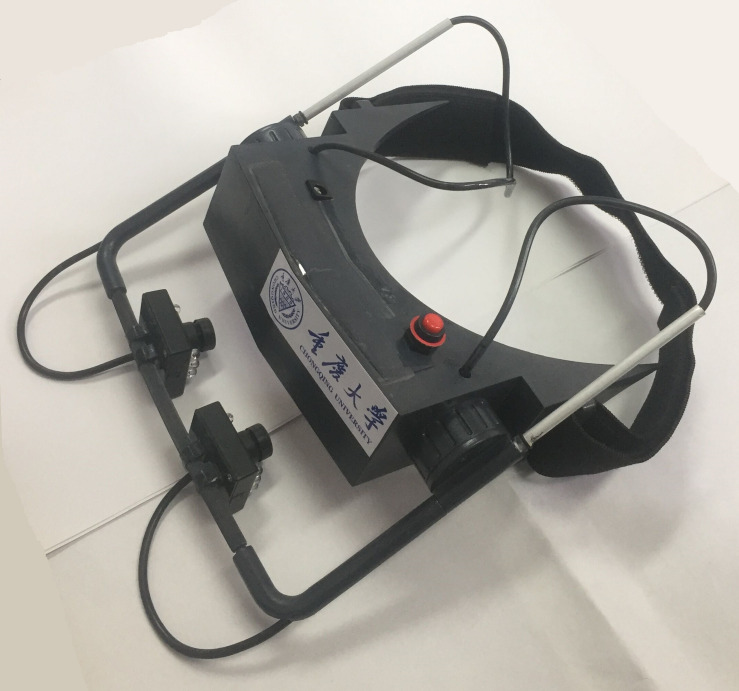
The eye tracker in our laboratory.

In recent years, with the development of computer video technology, cameras have become capable of recording the whole process of eye movement, extracting pupil information accurately and obtaining eye movement features for subsequent research. Therefore, an eye tracker has become the main means of obtaining eye movement information. Figure 1 shows the infrared video eye tracker developed by our laboratory. The device employs a video camera with an infrared LED to obtain eye movement images and transmit to the computer in real time. The software on the host can produce various patterns to guide the eye movement according to the experimental scheme so as to induce some diseases to be shown in the process of eye tracking.

In order to carry out medical research through eye movement information, a variety of experimental schemes need to be designed. Moving objects with different speeds and paths or images with specific scenes are generated on the computer screen to stimulate eye tracking and analyze its medical meaning. Our eye tracker supports gaze test, saccade test, smooth pursuit test, optokinetic test, positional test, and positioning test, and each can be selected for different purposes.

**FIGURE 2:**
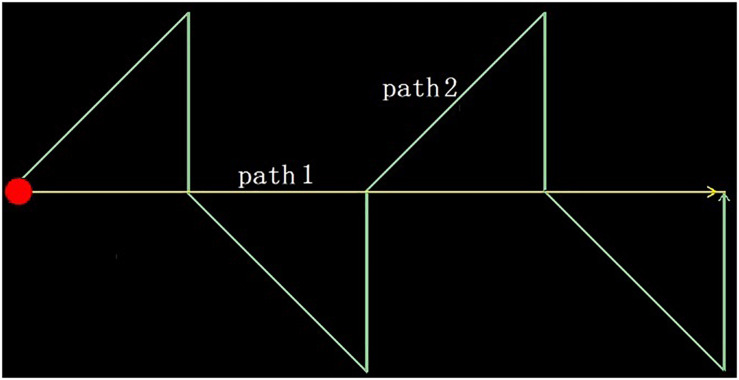
The moving trajectories of the light spots in the two experimental schemes.

In this study, we designed two experimental schemes: an optokinetic test and a smooth pursuit test. The tests are characterized by a spot of red light moving along a specific trajectory to guide the subjects’ eye tracking. In the first scheme, the light spot moves from left to right along a horizontal line in the center of the screen. After it reaches the right edge, it returns to the left and repeats the movement. In the latter scheme, the light spot moves along a two-way zigzag track, and after reaching the right endpoint, it returns to the left and starts again from the beginning, as shown in Figure 2. During the test, the subjects wore the eye tracker, sat in front of a screen, and were asked to track the moving light spots on the screen. At the same time, a video of the eye movement is transmitted to a host computer through the WiFi module of the eye tracker and is recorded on the same computer as that generating the tracking image. When the designated number of frames is recorded, the data acquisition ends.

#### The Original Eye Movement Features Extraction

**FIGURE 3:**
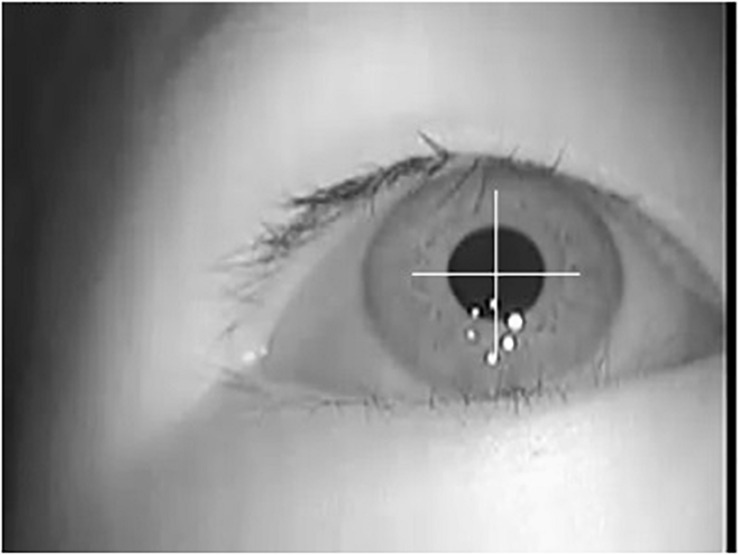
Pupil extraction of eye movement image.

Because the eye movement is obtained with infrared LED illumination, the light is more robust to interference and pupils are made more obvious and easy to extract. By using the general image analysis method, we can get accurate pupil information, as shown in Figure 3. According to the area covered by the detected pupil pixels, the position, area, shape, and other parameters of pupils in each frame of the video can be calculated as eye movement features.

In our study, based on the detected pupil area, we calculate nine parameters as optional features: position (including abscissa and ordinate), area (including pupil area and the area of the minimum bounding rectangle), symmetry (including the azimuth angle and the aspect ratio of the minimum bounding rectangle), and shape regularity (including the length of pupil outline, left to right area ratio, and top to bottom area ratio).

Due to the differences in pupil size, initial position, and view angle of the subjects, it is necessary to normalize the pupil information. Supposing *f*_*i*_is a certain pupil feature obtained from the eye movement image of frame*i*, and the video contains *M* frames in total, then the normalized feature *g*_*i*_ is obtained by Equation (1).

(1) $$g_{i}=\left(f_{i}-\underset{i=1∼M}{\text{min}}\left(f_{i}\right)\right)/\left(\underset{i=1∼M}{\text{max}}\left(f_{i}\right)-\underset{i=1∼M}{\text{min}}\left(f_{i}\right)\right)$$

With the above-mentioned processing, the values of the normalized features will be in the range of 0–1. *g* = [*g*_1_,*g*_2_,…,*g*_*M*_] is constructed as a feature vector. This feature corresponds to a certain parameter of the pupil in a specific experimental scheme. If there are *s* experimental schemes, and *k* parameters from the detected pupil are extracted in each scheme, then *s* × *k* feature vectors will be obtained for every subject; that is, *s* × *k* feature vectors will be established for each participant for subsequent classification and recognition. Because the feature ***g*** comes from pupil information directly, it is called an original eye movement feature in the following sections of this paper.

### Evolutionary Features Extraction

The characteristic information of eye movement obtained from the eye tracking experiment is time-varying, and its key pathological information may exist in some special period. Therefore, a disease classification model should be constructed based on eye movement features, and it needs to have memory of the temporal domain and should automatically retain important momentary information according to the rules or classification results. LSTM is a kind of special RNN that can handle long-distance dependence, which RNNs generally cannot manage, and is especially suitable for feature extraction and classification of time series signals. It is widely used in natural language processing, text analysis, speech recognition, machine translation, and other fields.

**FIGURE 4:**
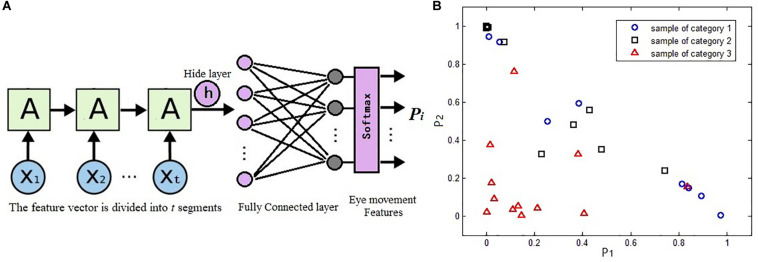
Long short-term memory (LSTM) network classification results served as evolutionary eye movement features: **(A)** the LSTM classifier in our work and **(B)** sample distribution map based on evolutionary eye movement features.

Because eye movement information can also be used as time series signals, the LSTM network is adopted to classify the eye movement features in our study. According to the eye tracking experiment schemes, the time sequence features *g* = [*g*_1_,*g*_2_,…,*g*_*M*_] obtained during the test should be divided into equal time slices and then input to each cell of the LSTM network. The last hidden layer of the LSTM is followed by a full connection network. The number of neurons in the output layer of the full connection network represents the classification categories. Finally, the discrimination result of the sample is obtained through a Softmax function, as shown in Figure 4A.

In our study, feature vector ***g***, which is used as the input of LSTM network, is called the original eye movement feature, and its classification ability is weak. It can be expected that some features may not be associated with partial specific diseases and thus have no contributions for classification. It is necessary to fuse all the features together effectively to get more accurate classification results.

Therefore, we use the classification results *P*_*i*_ of the LSTM classifier as evolutionary eye movement features for subsequent classification. In this study, we selected three categories, including healthy people, brain injury patients, and vertigo patients, so we will get *P*_*i*_,*i* = 1,2,3. Because *P*_1_ + *P*_2_ + *P*_3_ = 1, there are only two free variables in the three features; thus, we choose *P*_*1*_ and *P*_*2*_ as evolutionary features; that is to say, through the LSTM classification network, the original feature vector ***g*** with *M* elements is transformed into the evolutionary feature vector *Ef* = [*P*_1_,*P*_2_] with two elements. For a total of *s* × *k* features, we form *s* × *k* feature vectors named *Ef*. Because the evolutionary features are obtained by the LSTM network through learning from the labels of the training samples, they have stronger abilities to discriminate the samples.

Figure 4B presents the distribution of a sample set with a certain feature vector of *Ef*. The sample set includes 32 samples of three types, including 8, 12, and 12 samples of each type. It can be seen that the classification ability of this feature vector to these three categories of samples is poor.

### Disease Classification Method Based on Decision Tree and Random Forest

Because a single LSTM classifier comes from the eye movement feature of a certain experimental scheme, its classification ability for different diseases will vary greatly and cannot be accurately estimated. In fact, as shown by the previous analysis, assuming that there are *s* experimental schemes and *k* parameters are extracted from the detected pupil in each scheme, therefore *s* × *k* original features will be obtained for the subject. Because two feature components as [*P*_1_,*P*_2_] can be obtained through the LSTM network from each original feature, a total of 2 × *s* × *k* evolutionary features can be obtained for each subject. These features can be used to construct more effective classifiers.

Decision tree and RF algorithms are a kind of AI method that can effectively deal with such problems. RF refers to a classifier that uses multiple trees to train and predict samples. This classifier was first proposed by Leo Breiman and Adele Cutler and is registered as a trademark. RF is essentially a type of ensemble learning, which is a branch of machine learning. Its basic unit is a decision tree, and its output category is determined by which output category contains a majority of all the decision trees. Therefore, the construction of decision trees is the key step of an RF algorithm. In our study, decision tree and RF algorithms are used to solve the joint classification problem of eye movement features.

#### Decision Tree Construction

Decision tree is a supervised machine learning method. Given 2 × *s* × *k* evolutionary eye movement features of the sample and its label, a decision tree can be obtained by learning, which can give classification results to new samples. The decision tree generation algorithms include ID3, C4.5, and CART. The decision tree has a tree-like structure in which each internal node represents a feature judgment, each branch represents the output of a judgment result, and finally, each leaf node represents a classification result.

There are three categories of samples recruited in our study, and each sample has 2 × *s* × *k* features. The C4.5 algorithm is used to construct the decision tree. Generally, assume a sample set named as D, which contains *W* categories with *N* samples in total. *N*_*1*_, *N*_*2*_, … and *N*_*W*_ are the numbers of samples corresponding to *W* categories, and the equation *N* = *N*_1_ + *N*_2_ + ⋯ + *N*_*W*_ is satisfied. The number of features is named as *M*, *M* = 2 × *s* × *k*. The key tasks during construction of decision trees are the feature selection of the branch node and the feature value determination for comparison. Because *N* samples is assumed in the sample set, each feature has *N* values. There may be *N*−1 segmentation points to separate the values of a feature. For *M* features, there are *M* × (*N*−1) segmentation points. According to the C4.5 algorithm, the information gain rate should be calculated for each segmentation point.

First, the entropy of sample set D, called *H*(*D*), is computed in Equation (2):

(2) $$H\,\left(D\right)=-\sum_{l=1}^{W}\frac{N_{l}}{N}\,\log_{2}\frac{N_{l}}{N}$$

Let a feature segmentation point be *J*, which divides sample set D into two sets, *J*_*1*_and *J*_2_ = *N*−*J*_1_are the numbers of samples in the two sets, respectively. Considering the classification of the *J*_*1*_samples in the first set, suppose that it contains *p* categories,*p*≤*W*. Meanwhile, the numbers of samples of the *p* categories are named as *d*_1_,*d*_2_,…,*d*_*p*_, respectively, and *J*_1_ = *d*_1_ + *d*_2_ + … + *d*_*p*_, then the entropy of the first set is determined by Equation (3).

(3) $$H\,\left(J_{1}\right)=-\sum_{l=1}^{p}\frac{d_{l}}{J_{1}}\,\log_{2}\frac{d_{l}}{J_{1}}.$$

Similarly, considering the classification of the *J*_*2*_ samples in the second set, suppose that it contains *q* categories, *q*≤*W*, and the numbers of samples of the *q* categories are named as *d*_1_,*d*_2_,…,*d*_*q*_, respectively, and *J*_2_ = *d*_1_ + *d*_2_ + … + *d*_*q*_, then the entropy of the second set is determined by Equation (4).

(4) $$H\,\left(J_{2}\right)=-\sum_{l=1}^{q}\frac{d_{l}}{J_{2}}\,\log_{2}\frac{d_{l}}{J_{2}}$$

The conditional entropy of the sample set D segmented by feature *J* is calculated by Equation (5).

(5) $$H\,\left(D|J\right)=\sum_{i=1}^{2}\frac{J_{i}}{N}\,H\,\left(J_{i}\right).$$

According to the above-mentioned computation, the information gain *g*(*D*,*J*) of feature segmentation point *J* can be obtained [see Equation (6)].

(6) g⁢(D,J)=H⁢(D)-H⁢(D|J)

For the two sample sets formed according to the partition by segmentation point *J*, the entropy is determined by Equation (7).

(7) $$H\,\left(J\right)=-\sum_{i=1}^{2}\frac{J_{i}}{N}\,\log_{2}\frac{J_{i}}{N}$$

Finally, according to Equation (8), we can calculate the information gain rate *g*_*r*_(*D*,*J*) induced by dividing sample set D by feature segmentation J

(8) $$g_{r}\,\left(D,J\right)=g\,\left(D,J\right)/H\,\left(J\right)$$

After the information gain rates of all possible partition points *J* are calculated, the segmentation point *J* with the maximum information gain rate is selected to realize node branching. According to the constraints of decision tree construction, such as the tree depth, the minimum nodes allowed to be split, etc., the decision tree can be constructed. Generally, the decision tree will grow until a single category is reached; that is, *p* or *q* is 1. The decision tree can be used to pre-classify the test samples.

#### Random Forest Construction for Disease Classification

The so-called RF is used to randomly select samples and features, constructing multiple decision trees. The final classification results are determined by the voting of these decision trees. In order to enhance the robustness and avoid overfitting, there will be two random selections while constructing each decision tree. The specific steps are described as follows:

(1) First, the samples are randomly selected. For *N* samples of set D, the bootstrap method is used to randomly select *N* samples with replacement to form a sample set, which is used for the new construction of the decision tree.
(2) A total of *log*_2_([*M*(*N*−1))] feature segmentation points are randomly chosen from the *M* × (*N*−1) segmentation points at each branch node of the tree. The information gain rate of all the selected points are computed, and the segmentation point with the maximum value is selected to branch nodes. Meanwhile, the adopted segmentation point is marked to avoid being selected again at the following branch nodes.
(3) Each tree grows to the maximum without any pruning to get the decision tree.
(4) Repeat the above-mentioned steps (1) to (3), wherein multiple decision trees are generated to make up the RF. Use the RF classifier to test the new data. The classification result depends on the number of votes of the decision tree classifiers. The samples which have not been selected in the process of constructing the decision tree can be used as test samples to verify the performance of the RF classifier.

## Results

### Sample Grouping and the Original Eye Movement Features Extraction

In order to verify the classification method proposed above, we designed two experimental schemes, those being the optokinetic test and the smooth pursuit test; thus, *s* = 2. The moving trajectories of the light spots are shown in Figure 2. The subjects track the moving light spots while 250 frames of video are recorded at a frame rate of 30 frames per second. The pupil is detected from each frame, and then the abscissa *x*, ordinate *y*, and area *r* of pupil are extracted as eye movement features, *k* = 3. Therefore, the number of original eye movement features is *s* × *k* = 6.

In cooperation with medical institutions, we recruited 60 patients from two categories to participate in the experiment, including 24 patients with brain injury and 36 patients with vertigo. In addition, 36 healthy volunteers were invited for comparative testing. The healthy controls, brain injury (including cerebral infarction) patients, and vertigo patients are recorded as C1, C2, and C3, respectively.

According to the demands of classifier construction in our study, all 96 samples were divided into three groups. The first and the second groups were called training groups; the first group was used to train the LSTM classifier, and the second one was used to train the decision tree and RF. The third group was called the testing group and was used to test the performance of all intermediate or final classifiers. Each group contained 32 samples, and the distribution of each group of samples in each category is shown in Table 1.

**TABLE 1:** Composition of the three groups of samples.

	**C1**	**C2**	**C3**
Group 1	12	8	12
Group 2	12	8	12
Group 3	12	8	12

All 96 subjects participated in the eye tracking experiments. Six eye movement features as *g*_*i*_,*i* =  1,2,…,6 were calculated for each subject, and these features are called original eye movement features.

### LSTM Classifiers Training and the Evolutionary Features Extraction

Six original eye movement features were obtained in the previous step. Because they are derived from 250 frames of recorded eye movement, each feature is a vector containing 250 elements. The LSTM classifiers are constructed for each feature vector, and all six classifiers adopt the same structure. The 250 elements are divided into 10 slices according to the time sequence, and each slice contains 25 elements; that is to say, in Figure 4A, the vector Xi contains 25 elements, while *t* = 10. The number of neurons in the hidden layer is set to 64, and the output of the full connection network is three, corresponding to the sample categories.

**FIGURE 5:**
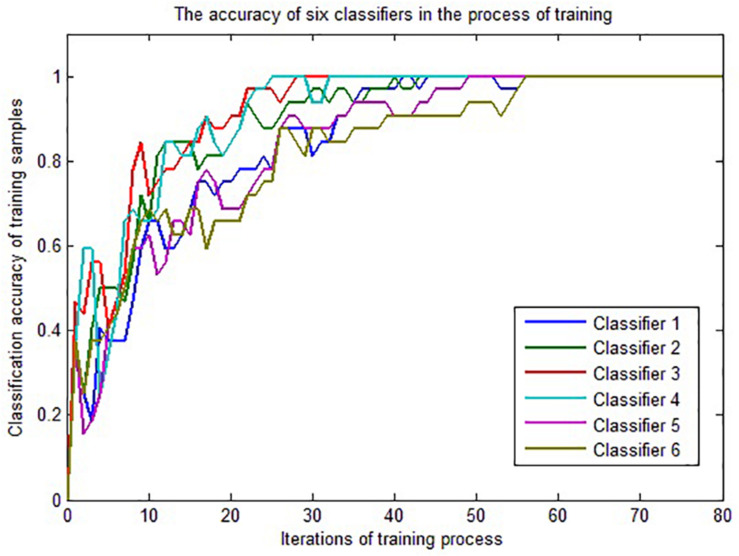
The classification accuracy changed in the process of training.

The 32 samples of group 1 are used for LSTM classifier training. Because one LSTM classifier is trained for each feature vector, six LSTM classifiers can be obtained. Python + Tensorflow is applied to realize the experimental simulation. Figure 5 shows the change of classification accuracy for the six classifiers over the rounds of iterations in the training process. The accuracy is defined as the ratio of the number of correctly classified samples to the total number of samples in all categories.

It can be seen from Figure 5 that the convergence rates of the six classifiers are different, but after nearly 60 iterations, all classifiers can correctly classify the training samples, so we get six LSTM classifiers based on the samples of training group 1.

In order to obtain the evolutionary eye movement features, we use the six LSTM classifiers to test the samples in training group 2. The sample features selected during the test of each classifier should correspond to the features adopted during the training of each classifier. The accuracy of each classifier is shown in Table 2. For each sample in training group 2, six vectors of *Ef=[P*_1_,*P*_2_] can be obtained from the test results of the six LSTM classifiers; that is to say, two feature components called *P*_1_and *P*_2_are obtained from each LSTM classifier. Eventually, 2 × *s* × *k* = 12 features are obtained for every sample. They are called evolutionary eye movement features and are expressed as*f*_1_,⋯,*f*_12_. Their corresponding relations are shown in Table 3.

**TABLE 2:** Accuracy of the six long short-term memory classifiers to the samples in training group 2.

	**Classifier 1**	**Classifier 2**	**Classifier 3**	**Classifier 4**	**Classifier 5**	**Classifier 6**
Accuracy (%)	68.75	62.5	87.5	84.375	71.875	78.125

**TABLE 3:** Corresponding relations among the evolutionary features and the test results of the long short-term memory classifiers.

	**Classifier 1**		**Classifier 2**		**Classifier 3**		**Classifier 4**		**Classifier 5**		**Classifier 6**	
Test result	*P*_*1*_	*P*_*2*_	*P*_*1*_	*P*_*2*_	*P*_*1*_	*P*_*2*_	*P*_*1*_	*P*_*2*_	*P*_*1*_	*P*_*2*_	*P*_*1*_	*P*_*2*_
Evolutionary features	*f*_*1*_	*f*_*2*_	*f*_*3*_	*f*_*4*_	*f*_*5*_	*f*_*6*_	*f*_*7*_	*f*_*8*_	*f*_*9*_	*f*_*10*_	*f*_*11*_	*f*_*12*_

The 12 features of Table 3 are evolutionary features, which are obtained by the six LSTM classifiers and the samples in training group 2. These features will be used to construct the decision trees and RF.

### Decision Tree Construction and Performance Analysis

After the 12 evolutionary eye movement features *f*_1_,⋯,*f*_12_are obtained, they can be adopted for the construction of decision trees and RF according to C4.5 algorithm.

**FIGURE 6:**
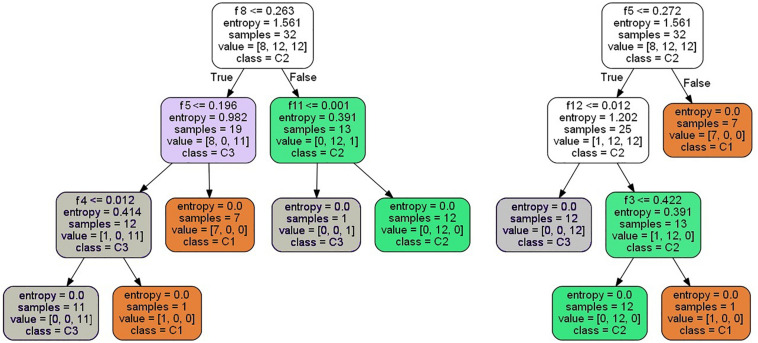
Two decision trees constructed by randomly choosing the samples and the features.

Because there are 32 samples in training group 2, there will be a maximum of 31 segmentation points for each feature; that is, *M* = 2 × *s* × *k* = 12, *N* = 32. Therefore, the total of feature segmentation point *J* is *M* × (*N*−1) = 372. The 32 samples are randomly selected 32 times by the bootstrap method to get a new sample set. Then, from the 372 segmentation points, *log*_2_⁡(*M*(*N*−1))≈8 feature segmentation points are selected randomly to construct a decision tree, which is then processed according to the steps in “Random Forest Construction for Disease Classification.” Figure 6 shows two decision trees built by the above-mentioned method.

It can be seen from the decision trees in Figure 6 that although eight features are selected, only three to four features are eventually adopted in each tree due to the small number of samples. If more samples are collected, each decision tree will adopt more features, and the classification robustness will be better.

### Construction and Performance Analysis of Random Forest Classifiers

The previous procedure is repeated to construct 20 decision trees based on the randomly selected samples and features. In the process of decision tree construction, the features used in the node branch are no longer selected. According to the classification results of each decision tree, the category with the most votes is regarded as the classification result of the subject.

Sample group 3 is especially designed for RF testing to analyze the performance of this method. For comparative analysis, sample group 3 is used to test all previous classifiers.

**FIGURE 7:**
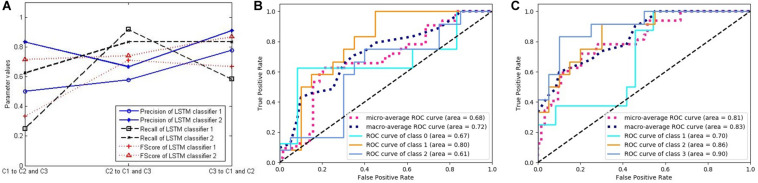
The performance of two long short-term memory (LSTM) classifiers. **(A)** Three classification performance indicators of two LSTM classifiers. **(B)** The receiver operating characteristic (ROC) curves and the area under the curve (AUC) values of LSTM classifier 1. **(C)** The ROC curves and the AUC values of LSTM classifier 2.

In order to verify the effectiveness of our method, we compute the three key indicators of precision, recall, and FScore to evaluate the performance of the classifiers. First, we choose two LSTM classifiers, and their results are presented in Figure 7. Figure 7A shows the above-mentioned three parameter values of LSTM classifiers 1 and 2. Because the experiment includes three categories, but these performance indicators are only defined for binary classification, we convert the three categories into three binary classification problems by selecting one category and evaluating it against the remaining two categories to calculate the parameters. The three markers in the horizontal direction represent the classifications of C1, C2, and C3 against the remaining classes, respectively. The ordinate represents the calculation results of the three indicators of the two classifiers.

Figures 7B,C respectively, correspond to two LSTM classifiers, each showing five curves. Three of them are ROC curves relating to the above-mentioned three binary classification problems, and the other two are the average values obtained by adopting macro- and micro-methods from the three curves. In addition, the area under the curve (AUC) values were calculated for each of the five curves, which also represent the classification performance.

**FIGURE 8:**
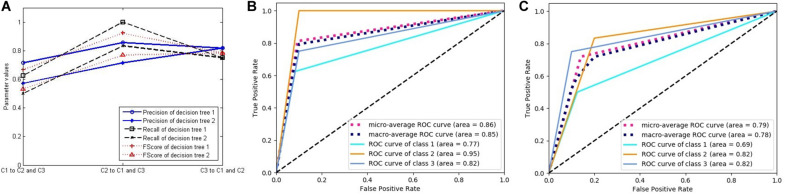
The performance of two decision trees constructed by C4.5 algorithm. **(A)** Three classification performance indicators of two decision trees. **(B)** The receiver operating characteristic (ROC) curves and the area under the curve (AUC) values of decision tree 1. **(C)** The ROC curves and the AUC values of decision tree 2.

Figure 8 was generated using the same method as in Figure 7, and each curve is associated with two classifiers. Figure 8 relates to the two decision trees constructed by the C4.5 algorithm. Because the decision tree gets the final decision result, the classification probability of the sample is only 0 or 1, with no intermediate value, such that there is only one threshold when constructing the ROC curve. Therefore, the ROC curve is composed of two line segments. In spite of this, the AUC values still reflect the performance of the classifiers.

**FIGURE 9:**
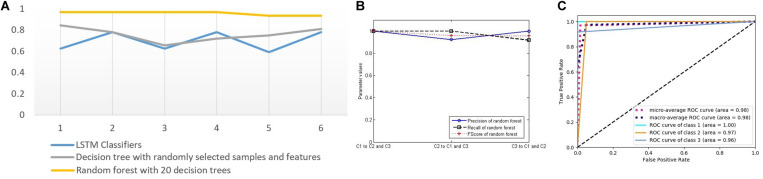
**(A)** Test accuracy of four classifiers for sample group 3 (six points on the horizontal axis represent six tests). **(B)** Three classification performance indicators of random forest. **(C)** The receiver operating characteristic curves and the AUC values of forest.

From these figures, we can see that the performance of the LSTM classifier is not stable. The performance of the different classifiers is very different, and the classification performance of the same classifier for different classes is also very different. Because the LSTM classifier comes from different features and some features are not related to some diseases, it has no classification value. The decision trees in Figure 8 come from the optimal selection of various evolutionary features with stronger classification abilities, which can be applied for the construction of RF. In order to verify the effectiveness of RF for disease classification, we compared the accuracy of the three classifiers with that of RF (see in Figure 9A). It contains three curves. The first one is the test results of the six LSTM classifiers, and the second one is the test results of six decision trees constructed by C4.5 algorithm. Finally, the last curve is the classification result of RF constructed by 20 decision trees. In order to facilitate the comparison and reduce the accidental factors, we also construct six RFs and record the classification accuracy for each one. Figure 9A shows the classification accuracy of the three classifiers for the six tests of sample group 3.

It can be seen from Figure 9A that the three classifiers have different accuracy rates for the testing samples. The accuracy rates of the six RFs are 0.96875, 0.96875, 0.96875, 0.96875, 0.9375, and 0.9375. In the six tests of 32 samples in the testing group, the numbers of correct classification are 31, 31, 31, 31, 30, and 30, which are significantly higher than that of other classifiers, showing good disease classification performance.

In order to further evaluate the disease classification ability of RF, we compute the three key indicators of precision, recall, and FScore to evaluate the performance (Figure 9B), ROC curves, and AUC values (Figure 9C). They all show better performance than that of other classifiers.

## Discussion

Medical research has confirmed that eye movement information is related to a variety of mental activities and physical diseases. However, due to the mechanism being unclear, it is difficult to directly associate eye movement characteristics with disease discrimination. Therefore, medical diagnosis methods based on eye movement are not practical, and it is especially difficult to distinguish multiple diseases. The aim of this study is to use advanced AI technology to extract more valuable eye movement features by a supervised learning method. To establish the relationship between diseases and eye activities, eye movement information is explored for effectiveness in relation to disorders under various experimental schemes. More effective disease classifiers can be constructed to mine the potential value of eye movement research.

The advantage of this research is that, by exploiting the learning ability of an AI algorithm for samples, the features can be automatically evaluated and chosen without prior pathological knowledge. The effective relationships can be established through experimental schemes for automatic extraction of eye movement features and disease classification.

The drawbacks of this study lie in several aspects: First, during the test, the subjects are required to stay awake, participate in the test as required, and try to keep their heads still, which limits the scope of use. Second, there are too few samples, resulting in the classifier not being sufficiently robust. In future work, we will collect more samples for training classifiers to improve the reliability. Based on research of pathological knowledge, we can present a more valuable experimental scheme and extract more features for disease discrimination. Advanced classification and clustering techniques will be explored to improve the ability of classifiers to distinguish specific diseases and may even discover some unknown diseases.

## Conclusion

To exploit eye movement information for disease diagnosis, AI technology is applied for self-learning and feature extraction. Features can be evaluated and automatically selected by a supervised learning algorithm. A variety of experimental schemes were designed to guide the subjects for eye tracking while a video of eye movement is recorded. The pupil is detected through image processing, and a variety of original eye movement features are obtained. For each original feature, an LSTM classifier is established, and the classification results are treated as evolutionary features, which are applied to build decision trees and an RF. The process of constructing the RF reflects the effectiveness of evaluation and selection of eye movement characteristics guided by the sample labels. The experimental results also demonstrate the efficiency of this method and greatly encourage the research value and prospect of AI techniques in disease diagnosis.

## Data Availability Statement

All datasets presented in this study are included in the article/supplementary material.

## Ethics Statement

The studies involving human participants were reviewed and approved by the Administrative office of Chongqing University Hospital. The patients/participants provided their written informed consent to participate in this study.

## Author Contributions

YM and LL designed the research. XC performed the research. LL and YH collected and analyzed the data. YM and YH wrote the manuscript. All authors contributed to the article and approved the submitted version.

## Conflict of Interest

The authors declare that the research was conducted in the absence of any commercial or financial relationships that could be construed as a potential conflict of interest.
